# Role of corporate governance in thriving green innovation practices: A case of emerging economy

**DOI:** 10.1371/journal.pone.0342897

**Published:** 2026-02-12

**Authors:** Unbreen Arif, Muhammad Bilal Ahmad, Aimun Khawaja

**Affiliations:** 1 Division of Management & Administrative Sciences, UE Business School, University of Education Lower Mall Campus, Lahore, Pakistan; 2 Hailey College of Banking & Finance, University of the Punjab, Lahore, Pakistan; 3 Minhaj University, Lahore, Pakistan; University of Central Punjab, PAKISTAN

## Abstract

In recent years the concept of green innovation has gained global attention in solving environmental issues and advancing sustainable development but opting for green innovation has always been challenging for firms due to the lack of incentives and negative externalities. Corporate governance refers to processes and systems to control the firm and play a crucial role in the decision-making process. The current study aims to explore the role of corporate governance in thriving dynamic capabilities of firms to adopt green practices to create competitive advantage, value and sustainable growth. The company-wise data of emerging economy were extracted from their financial and sustainability reports w.e.f 2014–2023 to analyze the nexus between corporate governance and green innovation practices. The empirical analysis was performed with the generalised method of moments (GMM) using stata and for text analysis python libraries were used to analyze governance mechanisms in thriving green innovation and its impact on financial performance. The results indicate that corporate governance has a significant influence on firm performance and green innovation mediates the relationship between corporate governance and firm performance. The result of the study will be useful for policymakers and stakeholders to implement good corporate governance practices to thrive for green innovation and to be competitive to earn sustainable earnings.

## Introduction

The escalating global environmental challenges such as climate change, resource depletion, and pollution highlighted the significance of opting for sustainable practices for corporations. This shift is driven by multiple stakeholders, including governments, consumers, investors, and civil society organizations, demanding increased environmental accountability. Technological advancements are playing a pivotal role in facilitating the transition toward a more environmentally conscious economy. Globally, sustainable economic models are expanding, with estimates projecting financial opportunities of up to $12 trillion annually by 2030 [1].

In response, firms are increasingly recognizing the strategic value of green innovation defined as the development of new or significantly improved products, processes, marketing methods, or organizational structures that yield both economic and environmental benefits. Once perceived largely as a regulatory burden, green innovation is now embraced as a strategic resource that enhances operational efficiency, reduces ecological impact, and fosters profitability and market expansion [2].

This issue is particularly relevant for emerging economies, where rapid industrialisation, urbanization, and resource constraints create unique environmental pressures [3,4]. At the same time, these markets also offer significant opportunities for the adoption of green technologies and sustainable products [5]. In such contexts, green innovation becomes not only an environmental imperative but also a vital economic driver [6]. However, realizing its potential depends heavily on a supportive organizational setting, which in turn is shaped by effective corporate governance.

Corporate governance provides the accountability, incentives, and monitoring structures necessary to cultivate innovation. Well-designed governance systems promote transparency, align managerial decisions with long-term objectives, and improve risk oversight [7,8], enabling firms to channel resources toward sustainable value creation [9]. Prior studies indicate that governance fosters innovation by strengthening accountability [10], facilitating risk taking in strategic decisions [11] and supporting investments in green R&D [12,13]. Board diversity, shareholder activism, and performance-based incentives for executives have also been shown to encourage stronger innovation outcomes [14]. On the other hand, excessively rigid governance systems or conservative risk preferences may suppress creativity and reduce innovation potential [15]. Governance mechanisms have also been linked to greater transparency and resource efficiency [16]. These mixed findings suggest the relationship between governance and innovation is not straightforward and requires deeper exploration, particularly in the realm of sustainability-focused strategies.

This study draws on Stakeholder Theory [17] to examine this nexus. which provides a compelling lens to examine the corporate governance and green innovation nexus. The framework emphasizes that firms are accountable not only to shareholders but also to a wide range of stakeholders, including employees, consumers, suppliers, local communities, and the environment. Accordingly, decisions regarding environmental investment are shaped as much by stakeholder demands as by profitability concerns. The theory also sheds light on agency conflicts that arise from diverging stakeholder interests on environmental issues, underscoring the role of governance in embedding sustainability into corporate strategy [7]. The earlier research based on Stakeholder Theory examines either the effects of corporate governance on environmental outcomes or on firm performance [13,18–20], without necessarily considering that green innovation is part of the process by which firms respond to stakeholder pressures. This study extends Stakeholder Theory through the conceptualization of green innovation as a stakeholder-driven transmission mechanism for aligning corporate governance with long-term financial performance on environmental accountability within the constraints of emerging markets.

This study empirically investigates the nexus between corporate governance, green innovation, and firm financial performance in context of emerging economy of Pakistan. The country faces significant environmental challenges, from deforestation and pollution to heightened climate vulnerability [21,22]. At the same time, its increasing policy focus on sustainability, evident in national environmental strategies and international commitments, makes it a relevant case for studying how governance structures can shape green innovation and financial outcomes.

Although scholarship on this subject is expanding, much of the evidence has been drawn from developed economies. Research in emerging contexts remains scarce, despite the fact that weaker institutions, less mature regulatory frameworks, and distinctive stakeholder pressures may fundamentally alter governance–innovation dynamics. Moreover, while governance has been linked separately to innovation or performance, the mediating role of green innovation in translating governance practices into financial success remains largely underexplored.

Addressing these gaps, this study makes three contributions. First, it empirically investigates the relationship between corporate governance, green innovation, and financial performance in the context of an emerging economy. Second, it examines the mediating role of green innovation, thereby extending Stakeholder Theory by clarifying how governance mechanisms influence both environmental and financial outcomes. Third, by focusing on Pakistan, the study provides context-specific insights relevant for policymakers, investors, and managers designing governance frameworks to stimulate sustainable innovation.

The remainder of this paper is structured as follows: Section 2 reviews the literature and develops the hypotheses. Section 3 outlines the methodology and data sources. Section 4 presents empirical results. Section 5 discusses findings in relation to existing research. Section 6 concludes with implications for policy and future research.

## Literature review

Corporate environmental compliance has grown into a strategic imperative in the face of the global energy crisis and climate change. Compliance can not only reduce regulatory pressure [23], while also encourages sustainability-oriented solutions, enables green innovations [24] and support broader sustainable development [25]. Firms that actively comply with or even exceed environmental regulations are better positioned to minimize ecological harm, enhance resource efficiency, and generate innovative solutions. Strong compliance practices can also enhance corporate reputation, attract environmentally aware investors and customers, and reduce costs through operational efficiency. As environmental issues continue to escalate, environmental compliance is expected to become a central element of corporate strategies and regulatory approaches [26].

Traditionally, the firm’s role was framed around maximizing shareholder value, yet this view has evolved. Companies are increasingly recognized as socially responsible entities that must account for their impact on both stakeholders and the environment. Evidence shows that well-governed firms often engage in socially responsible activities and simultaneously achieve higher performance outcomes [27]. However, some findings remain contested; for instance [28] argue that CSR activities may, in certain cases, detract from a firm’s ability to maximize shareholder value. Adding to this debate, [29] highlights how corporate social responsibility, when embedded in competitive strategy and daily operations, functions as more than symbolic action, instead positioning itself as a long-term investment aligned with the United Nations’ Sustainable Development Goals (SDGs).

Corporate governance has long been considered central to shaping a firm’s financial outcomes. Empirical research highlights that ownership type and governance structures significantly influence performance [30], while factors such as board composition, CEO duality, and board size remain critical determinants [31]. External growth opportunities may further moderate this relationship, suggesting that governance-performance linkages cannot be understood in isolation [32].

Findings in the literature, however, remain mixed. Some studies report that better governance enhances credibility with shareholders, mitigates self-dealing, and improves profitability [33–35]. Similarly, governance mechanisms such as board independence and audit quality have been linked to financial performance across contexts [11,36,37]. Contradictions also appear in industry-specific contexts: independent directors and CEO duality improved construction industry returns [38] while audit quality had a negative effect on market performance [39].

Prior studies have long debated the extent to which governance mechanisms improve firm outcomes. Some scholars report that strong governance—through independent boards, disclosure practices, and accountability systems—has a positive effect on financial performance to [38,40,41]. However, evidence is far from conclusive studies such as [42,43] emphasize complexity and call for nuanced analysis. [7]. [44] demonstrate that in certain contexts, governance does not significantly contribute to profitability, suggesting that the relationship may be conditional or context dependent. Overall, the presence of mixed and context-dependent results implies that the relationship between governance and performance is not linear, particularly in emerging economies where institutional frameworks remain less developed. Based on these arguments, the first hypothesis is:

H1: Corporate Governance has an impact on corporate Financial Performance.

A growing body of literature highlights that governance can influence a firm’s ability to adopt and implement green innovation strategies. For instance, board expertise, gender diversity, and sustainability committees have been linked to stronger environmental initiatives and innovative practices [45–47]. Governance, in this sense, does more than monitor; it actively shapes the firm’s orientation toward sustainable practices. [48] further argue from a multi-level governance perspective that governance frameworks are critical in steering organizations toward sustainability transitions, while [29] emphasizes the role of CSR and social reporting in legitimizing firms’ environmental strategies. Similarly, top managers’ smog-risk perception enhances innovation when mediated by CSR engagement [49]. Shareholder activism can accelerate green innovation by improving decision-making efficiency [50], while corporate digitalization fosters innovation directly and indirectly via human capital [51,52]. Conversely, strict environmental penalties may reduce both the quality and quantity of innovation [53]. Together, these studies studies indicate that corporate governance plays a strategic role in shaping firms’ environmental orientation by influencing decision making structures, managerial incentives and stakeholder engagement. The governance mechanisms appear to actively enable or constrain firms’ capacity to pursue green innovation. A well-structured governance fosters green product, process, and image innovation. Therefore, this study proposes that effective corporate governance enhances the level of green innovation within firms.

H2: Corporate Governance influence corporate green Innovation.

Green innovation, encompassing eco-friendly products, processes, and systems, is increasingly viewed as both an environmental and competitive imperative. It has been defined as *“the development and implementation of new products, processes, or services that reduce environmental impacts and promote sustainable development”* [54,55]. While traditionally focused on environmental outcomes, recent scholarship extends green innovation to include social dimensions of sustainability [55,56]. Evidence shows that green innovation improves efficiency and reputation, thereby enhancing firm profitability [57]. Studies confirm significant positive impacts on financial indicators such as ROA, ROE, and Tobin’s Q [58–60]. However, the relationship is not uniformly positive; strict environmental contexts may suppress financial benefits, and results can vary by intensity of innovation and country-level conditions. Importantly, green innovation is not exclusive to large corporations—it also drives competitiveness among SMEs in developing economies [61].

While innovation has traditionally been measured in terms of R&D or patents, recent research stresses that green innovation covering product, process, and image dimensions provides firms with a competitive edge in sustainability-driven markets. Evidence suggests that green innovation can improve profitability and market valuation [58–60], Despite occasional non-linear or inconsistent results, the majority of evidence supports a positive link between green innovation and performance [62–65]. However, the impact is not uniform; resource constraints, regulatory pressures, and consumer expectations can mediate the extent to which green innovation translates into financial outcomes. In emerging economies, where institutional support for sustainability is weaker, the performance benefits of green innovation are particularly underexplored. On this basis, the third hypothesis of study is:

H3: Corporate green innovation has an impact on corporate financial performance.

Although prior studies confirm that governance enhances innovation and, separately, that innovation improves performance, few have explored whether green innovation serves as a mediating pathway between governance and firm outcomes. [66] reported green product innovation mediates between environmental ethics and competitive advantage, while green adaptive ability strengthens links between green human capital and product innovation [67]. Similarly, eco-innovation mediates the relationship between CSR and dynamic capabilities [68] and between green credit and performance outcomes in textiles [69].

The limited available evidence, largely from developed economies, indicates that governance influences financial performance indirectly by fostering innovation-oriented strategies [45,47]. Further evidence shows that climate risk reduces financial performance, but CSR mitigates this effect, particularly for accounting-based measures [70]. ESG practices also improve firm value, with green innovation acting as a full mediator [71]. Likewise, ESG reporting strengthens the credibility of green signals in outputs [72]. These findings collectively establish green innovation as a central pathway through which governance and CSR initiatives translate into financial performance.

Yet, in emerging economies, where governance systems are less mature, this indirect mechanism has not been sufficiently tested. This study extends the corporate governance, innovation and organizational performance literature by conceptualising innovation not solely as R&D input or technological advancement, but as a multidimensional construct encompassing green product, process, and image innovation. By integrating this comprehensive view of green innovation within Stakeholder Theory, the Governance, Innovation, Performance (GIP) Pathway is introduced for emerging economies. This pathway refines existing frameworks by highlighting that governance mechanisms influence not only technological R&D-based innovation but also sustainability-oriented innovations that legitimize firms in the eyes of diverse stakeholders. Unlike prior research conducted primarily in developed markets [26,45], our findings demonstrate that in institutional contexts characterized by resource constraints and weaker enforcement mechanisms, governance effectiveness materializes through the pursuit of green product, process, and image innovation, which in turn drive performance outcomes. This perspective extends existing theory by shifting the focus beyond simple confirmatory relationships, offering instead a more nuanced framework that situates legitimacy and sustainability imperatives at the core of the governance, innovation and performance relationship. Overall, the literature suggests that governance and innovation are closely connected and that innovation enhances firm performance. However, these relationships are largely examined in isolation. What remains unclear is whether governance improves financial outcomes directly or whether its effects materialize primarily through sustainability-oriented innovation. This unresolved mechanism is particularly salient in emerging economies, where governance structures may not immediately translate into financial gains but instead operate by enabling legitimacy enhancing green innovations. Addressing this gap, the present study proposes that green innovation mediates the relationship between corporate governance and firm performance, such that governance contributes to performance primarily by enabling sustainability-driven innovations

H4: Green innovation mediates the relationship between corporate governance and financial performance.

The review reveals three major gaps. First, the literature offers mixed evidence on whether governance directly improves firm performance, especially in emerging markets. Second, while governance has been shown to encourage innovation, little is known about its role in driving green innovation that combines product, process, and image dimensions. Third, despite growing interest in sustainability, the mediating effect of green innovation in the governance–performance link has been largely overlooked, particularly in contexts where institutional enforcement and sustainability practices are evolving.

In addressing these gaps, the present study contributes in three important ways. One, it provides empirical evidence on how corporate governance affects both firm performance and sustainability-driven innovation in an emerging economy context. Two, it broadens the understanding of innovation by focusing not only on R&D but also on green product, process, and image innovations. Moreover, although research recognizes the mediating role of green innovation, little is known about how governance-driven green practices translate into financial outcomes in emerging economies, where institutional pressures, regulatory frameworks, and resource constraints differ from developed markets. The current study introduces and tests a mediation model to explain how governance translates into financial outcomes through green innovation, thereby offering new insights into how firms can align governance practices with sustainability goals to achieve competitive advantage.

## Methodology

The study examines corporate governance (CG), green innovation (GI) and firm financial performance (FP) using data from non-financial companies listed on the Pakistan Stock Exchange (PSX) between 2014 and 2023. The initial dataset comprised 70 firms that were part of the KSE- 100 Index at any point during this period, including both active and delisted entities. After excluding firms with missing corporate governance disclosures or incomplete financial information, the final sample comprised 38 firms, yielding 380 firm-year observations over ten years. The analysis focuses on the direct effect of corporate governance on financial performance, the direct effect of green innovation on performance, and the indirect role of green innovation as a mediating factor.

With a focus on the impact of corporate governance (CG) and green innovation (GI) on firm performance(FP), the panel dataset of emerging market firms was utilized to examine the direct and indirect impact of Green innovation on performance. The firm financial performance is the dependent variable the Corporate Governance is the independent variable and the green innovation’s direct and indirect effect with was examined by following the technique of [73,74] of the generalized method of moment (GMM) using stata17. The general equation is


$${FP}_{it}=\alpha \;{FP}_{i,t-\text{1}}+{CG}_{it}+{GI}_{it}+S_{it}+L_{it}+\varepsilon_{it}$$ (1)

Where ${FP}_{it}$ indicates firm performance at time t which is measured with ratio of return on asset (ROA), ${\;FP}_{i,\;t-\text{1}}$ is the one period lag operator, i.e., last year’s firm performance, ${CG}_{it}$ is the Corporate Governance variable, ${GI}_{it}$ is the green innovation, the variables $S_{it}$ and $L_{it}$ also incorporated as control variables [75] to get results. The biggest challenge in emerging economy is related to availability of ESG data [76]. As lack of regulatory framework and non standardized approach the compliance to ESG disclosures are voluntary. Similarly, R&D data is rarely disaggregated at the green innovation level across firms or sectors. To address the data availability challenge the application of Machine learning methods has provided a new and alternative technique to measure Corporate green innovation [77,78] by using topic modelling for text analysis. This method has the advantage of getting insight into firms’ dynamic research activities and innovative capabilities [79]. This method is widely used in literature to analyze disclosures [80,81]. The text related to firms’ green innovation is extracted from sustainability reports and annual reports through Machine learning by using python library of nltk. The text-based measure of green innovation is made through lexicon based approach of VADER and the sentiment of score is measured on predefined themes of green product and process innovation by using themes adapted from studies of [82]. The themes used “emission of hazardous substances or waste”, “recycles waste”, “consumption of water, electricity, coal, oil”, “materials of low pollution”, “consumption of least amount of energy, resources”, “product is easy to recycle, reuse, and decompose”. To reduce measurement noise, generic environmental terms unrelated to innovation activities were excluded, and overlapping or ambiguous terms were refined through iterative screening. The preprocessing of data text was a critical step, multiple data preprocessing techniques were used to get it prepared for analysis include tokenization, elimination of links, numeric, punctuations, stop words etc., change case to convert all text into small case and lemmatization. Tokenization is the process of splitting texts into strings. The sentiment Lexicon VADER was used by using python library. The green innovation index was computed as the frequency-weighted intensity of green innovation keywords scaled by total word count, ensuring comparability across firms and years. The index exhibited meaningful variation across firm-year observations and aligned with theoretical expectations. Further, the uses of publicly available annual and sustainability reports and the application of lexicon-based approach ensures reproducibility of index. To measure CG, multiple firm level attributes were used as Board Size (BS), Board independence (BI), Board Diversity (BD) CEO duality (CD) Number of board meetings in a year (BM), independence of audit Committee (AI). The corporate governance index was calculated by using firm level data and following studies of [83,84]. The firm financial performance is measured with ratio of earnings before interest and taxes to total assets.

To estimate the dynamic model, this study applies the generalized method of moments (GMM) introduced by [73]. The difference GMM approach transforms the model into first differences, which helps to control for unobserved fixed effects and potential endogeneity by using lagged values of endogenous variables as instruments. A two-step estimator was adopted, as it provides greater efficiency and remains robust in the presence of heteroscedasticity [85]. The approach relies on the assumption that lagged values of both exogenous and endogenous variables are valid instruments. It also requires that the first-differenced residuals do not exhibit second-order serial correlation, and that instruments remain uncorrelated with the error term Arellano & Bond (1991). To validate these conditions, Arellano–Bond tests for AR(2) and the Hansen/Sargan tests for over-identifying restrictions were conducted, ensuring the reliability of the instruments and the robustness of the estimated results.

## Results and discussion

The panel data was used to empirically investigate the role of CG, GI and FP. Before applying the GMM model, several preliminary checks were performed to ensure the data were suitable for analysis. To assess multicollinearity among the components of the Corporate Governance Score (CG), a linear regression was conducted using CG as the dependent variable (Table 1). Board size and CEO duality were excluded due to perfect collinearity. For the remaining indicators, the Variance Inflation Factor (VIF) were all below 2.0, indicating no serious multicollinearity. The composite corporate governance score (CG) was constructed by following prior research of [83,84].

**Table 1:** VIF analysis.

Variable	VIF	1/VIF
b_ind	1.36	0.735
b_div	1.06	0.904
b_meet	1.07	0.935
aud_ind	1.26	0.791

Table 2 presents the descriptive statistics and correlation matrix. The mean value of return on assets (RoA) is 2.06 with a standard deviation of 1.27, indicating moderate profitability variation across the firms. The average CG Score (0.54), indicating a moderate level of governance compliance. Green innovation(GI) has a relatively low (mean = 0.134), reflecting the early or limited adoption of green practices. Leverage (mean = 0.53), highlighting that on average, firms finance around half of their assets with debt, while firm size (measured in logarithmic form) has a mean of 2.36.

**Table 2:** Descriptive statistics and correlation matrix.

Variable	Mean	Std. dev.	FP	CG	GI	leverage
FP	2.06	1.27	1.000			
CG	0.54	0.13	0.183 **	1.000		
GI	0.134	0.653	−0.232***	0.063**	1.000	
leverage	0.53	0.21	−0.336***	−0.057***	0.035	1.000
size	2.36	0.106	−0.021	0.251***	0.139**	0.017

*** p < 0.01, ** p < 0.05, * p < 0.1.

The correlation matrix shows FP is negatively correlated with GI (r = −0.232, ***p < 0.01), indicating that higher levels of GI be associated with lower short-term profitability, potentially due to the upfront costs of innovation initiatives. Conversely, FP is positively correlated with CG (r = 0.183, **p < 0.05), suggesting that stronger corporate governance is associated with improved firm performance (FP). Leverage exhibited a strong negative relationship with FP (r = −0.336, *p < 0.01), reflecting the potential financial burden of debt on firm profitability. Among the independent variables, CG and size are positively correlated (r = 0.251, *p < 0.01), implying that larger firms tend to have stronger governance practices. Additionally, GI is positively correlated with size (r = 0.139, p < 0.05) and weakly correlated with leverage, while CG has a small but significant negative correlation with leverage (r = −0.057, *p < 0.01). All Variance Inflation Factors (VIFs) were below conventional thresholds, suggesting no multicollinearity concerns among the variables.

Pooled OLS regression results are presented in Table 3. Findings provide initial support for the study’s hypotheses. CG Score is significantly and positively related to FP across models, confirming the role of governance in enhancing FP. GI is negatively associated with FP, highlighting short-term performance costs of innovation. Leverage consistently exhibits a negative effect on FP, while firm size shows mixed but largely insignificant effects.

**Table 3:** Pooled OLS results.

Variable	Model 1	Model 2	Model 3	Model 4
RoA2				
cg_score	1.794*** (0.486)	−0.504* (0.265)		1.590*** (0.477)
innovation			−0.435*** (0.0931)	−0.405*** (0.0923)
leverage	−1.998*** (0.293)	0.0865 (0.160)	−2.014*** (0.290)	−1.963 (0.286)
size	−0.746 (0.595)	1.010*** (0.324)	0.178 (0.575)	−0.337 (0.588)
Constant	3.909*** (1.370)	−2.025*** (0.747)	2.767*** (1.366)	3.090** (1.351)
observations	380	380	380	380

*** p < 0.01, ** p < 0.05, * p < 0.1.

Panel regressions were conducted using both Fixed Effects (FE) and Random Effects (RE) models (Tables 4 and 5). The Hausman test (χ^2^ = 16.32, p = 0.01) confirmed that the FE model is more appropriate, accounting for unobserved firm heterogeneity. Under the FE model, GI remained negatively and significantly related to FP, while leverage exerted a strong negative impact. Firm size positively influenced FP under FE, suggesting that larger firms benefit from scale advantages. Results under RE were directionally consistent but exhibited some coefficient variations.

**Table 4:** Panel data regression model (FE).

Variable	Model 1	Model 2	Model 3	Model 4
RoA2				
cg_score	−0.437* (0.495)	−0.314* (0.369)		−0.489* (0.492)
innovation			−0.163*** (0.0723)	−0.166** (0.0724)
leverage	−2.289*** (0.459)	−0.338 (0.342)	−2.335*** (0.457)	−2.345*** (0.457)
size	3.697*** (0.913)	0.204 (0.681)	3.341*** (3.818)	3.731*** (0.908)
Constant	−5.225*** (2.041)	0.001 (1.522)	−4.574*** (1.919)	−5.225** (2.028)
observations	380	380	380	380

*** p < 0.01, ** p < 0.05, * p < 0.1.

**Table 5:** Panel data regression model (RE).

Variable	Model 1	Model 2	Model 3	Model 4
FP				
CG	0.142 (0.473)	−0.504* (0.265)		0.314* (0.471)
GI			−0.220*** (0.0743)	−0.220*** (0.0748)
leverage	−2.155*** (0.395)	0.086 (0.160)	−2.156*** (0.375)	−2.143*** (0.373)
size	2.131*** (0.791)	1.010*** (0.324)	1.884*** (0.701)	1.636** (0.750)
Constant	−1.909 (1.785)	−2.025*** (0.747)	−1.218 (1.658)	−0.809 (1.695)
observations	380	380	380	380

*** p < 0.01, ** p < 0.05, * p < 0.1.

To ensure the robustness of the analysis, preliminary checks were carried out for pooled OLS and panel regression models, with the results reported in Table 4. These diagnostics revealed the presence of both heteroskedasticity and autocorrelation in the dataset. Table 6 further summarizes the robustness tests, where the Wooldridge test confirmed significant first-order autocorrelation (F(1, 37) = 21.055, p = 0.01), while the modified Wald test pointed to groupwise heteroskedasticity. Given these issues, it became necessary to adopt a more advanced estimation approach. Accordingly, the Generalized Method of Moments (GMM) was employed to effectively account for endogeneity, heteroskedasticity, and autocorrelation.

**Table 6:** Autocorrelation and heteroskedasticity tests.

Variable	Test	Model	Statistic	Hypothesis	Results
ROA2	Heteroskedasticity	FEM	Prob>chi2 = 0.01 < 5%	Reject Ho	Yes
	Autocorrelation	FEM	Prob> F = 0.000 < 5%	Reject Ho	Yes
Innovation	Heteroskedasticity	FEM	Prob>chi2 = 0.000 < 5%	Reject Ho	Yes
	Autocorrelation	FEM	Prob> F = 0.01 < 5%	Reject Ho	Yes
ROA2	Heteroskedasticity	FEM	Prob>chi2 = 0.000 < 5%	Reject Ho	Yes
	Autocorrelation	FEM	Prob> F = 0.000 < 5%	Reject Ho	Yes
ROA2	Heteroskedasticity	FEM	Prob>chi2 = 0.01 < 5%	Reject Ho	Yes
	Autocorrelation	FEM	Prob> F = 0.000 < 5%	Reject Ho	Yes

Dynamic panel estimation was conducted using the two-step difference GMM approach, satisfying all necessary preconditions (Roodman, 2006). The results (Table 7) confirmed strong persistence in firm performance, with lagged RoA2 significantly predicting current FP. Governance reforms demonstrated delayed but positive effects on firm performance and innovation, consistent with the view that governance structures yield benefits over the longer term. Innovation, in turn, significantly enhanced financial performance, supporting its mediating role.

**Table 7:** Dynamic panel data estimation two-step difference GMM.

Variable	Model 1	Model 2	Model3	Model 4 (indirect)
	FP	GI	FP	FP
L1.	0.423*** (0.024)	−.157*** (0.017)	0.457*** (0.014)	0.415*** (0.0954)
CG				
--.	−0.997* (0.4712)	0.406 (0.820)		−0.576 (0.775)
L1.	−0.357 (0.278)	−.317 (0.40)		−.766 (0.749)
L2.	0.807** (0.363)	0.448*** (0.008)		0.896*** (0.557)
GI				
--			−0.778*** (0.022)	−0.107*** (0.032)
L1.			−0.074*** (0.025)	−0.066*** (0.023)
L2.			0.258*** (0.013)	0.238*** (0.027)
Size				
--.	2.288* (1.322)	0.281 (0.293)	1.602 (0.980)	1.724 (1.403)
L1.	1.419* (1.085)	−0.929 (0.674)	1.898*** (0.514)	2.135 (1.533)
L2.	−.405 (0.689)	0.309 (0.368)	−.714 (0.670)	−0.654 (1.136)
Leverage				
--.	−0.881* (0.441)	−0.254** (0.1200)	−0.963*** (0.330)	−1.412 (0.771)
L1.	0.411 (0.494)	−0.130 (0.188)	0.176 (0.432)	0.106 (0.788)
L2.	1.128** (0.510)	−0.182* (0.184)	1.369*** (0.385)	1.479 (0.692)
AR(1)	−2.53	−1.03	−2.56	−2.50
AR(2)	−0.89	−0.94	−0.82	−1.05
Hansen test	34.89	11.20	34.65	33.44
Sargan test	215.8	255.57	205.81	233.70

*** p < 0.01, ** p < 0.05, * p < 0.1.

Diagnostic tests validated the GMM specifications. The Arellano–Bond AR(2) tests confirmed no second-order autocorrelation, and Hansen tests supported instrument validity. Although Sargan tests were significant (as expected under heteroskedasticity), Hansen tests confirmed the robustness of the chosen instruments.

Diagnostic test for Model-1 confirm the robustness of the GMM specification. The Arellano-Bond AR(2) test indicated no second-order autocorrelation (*z* = –0.89, *p* = 0.375), satisfying a key condition for instrument validity. The Hansen test of overidentifying restrictions confirmed the appropriateness of the instruments (*χ*^*2*^(151) = 34.89, *p* = 1.000), while the non-robust Sargan test returned a significant result (*p* = 0.000), as expected under heteroskedasticity. Overall, the specification satisfies core GMM validity criteria.

To ensure the validity of the dynamic panel model-2 estimated through two-step difference GMM, several diagnostic tests were employed. Diagnostic tests confirm the robustness of the GMM estimates. The Arellano-Bond test for AR(2) errors was insignificant (*p* = 0.413), meeting the assumption of no second-order autocorrelation. Although the Sargan test suggested potential concerns (*p* = 0.002), the Hansen test (*p* = 1.000) confirmed the validity and exogeneity of instruments, supporting the overall reliability of the estimation. The Hansen test, which is robust to heteroskedasticity, was highly non-significant (*χ*^*2*^(151) = 34.65, *p* = 1.000), indicating that the instruments used in the model are valid and uncorrelated with the error term. Despite the large number of instruments, there is no indication of instrument proliferation weakening the test, as the Hansen statistic is well below the instrument count.

To validate the robustness of the two-step system GMM estimation in Model-3, several diagnostic tests were performed. The Arellano-Bond test for autocorrelation in first-differenced errors showed no significant first-order autocorrelation (*z* = –1.03, *p* = 0.303) or second-order autocorrelation (*z* = –0.94, *p* = 0.348). This confirms the absence of serial correlation in the error terms, satisfying a key assumption of GMM. The Hansen test of overidentifying restrictions produced a chi-square statistic of 11.20 (df = 158, *p* = 1.000), indicating that the instruments used were valid and uncorrelated with the error term. This suggests no evidence of instrument overfitting or endogeneity, despite a relatively large number of instruments. Although the Sargan test was significant (*χ*^*2*^(158) = 255.57, *p* < 0.01), it is sensitive to heteroskedasticity and not robust in two-step estimations, hence greater reliance is placed on the Hansen test. The results of Direct path analysis indicate:

H1 (Direct):CG → FP (supported)

H2 (Direct): GI → FP (supported)

H3 (Direct):CG → GI (supported)

To assess the significance of indirect effect, Sobel tests or bootstrapped confidence intervals are frequently used in conventional cross-sectional mediation analysis [86]. However, Sobel tests depend on the indirect effect’s normality, which might not hold true for small samples or panel data that exhibits heteroskedasticity and autocorrelation [86]. Further, Bootstrapping and Sobel tests cannot easily accommodate endogenous regressors or lagged instruments as used in GMM estimations [87,88]. This raises validity issues in instrument-based estimation frameworks. So, for mediation analysis a Barron and Kenny [89] approach was applied, this approach is methodologically compatible with GMM estimations [90]. In Table 7, Model 1 presents the results of direct impact of CG → FP. Results show a significant positive association (*β* = 0.807, *p* < 0.05), indicating that corporate governance practices directly impact firm financial performance. The positive impact of corporate governance on firms performance is supported by studies of [34,35].A statistically significant negative relationship at L1 indicates that an increase in current CG is associated with a decrease in FP. The results are align with the findings of [91]. This counterintuitive finding may imply potential short-term costs associated with implementing governance reforms or inefficiencies in governance practices. The size of the firm statistically insignificant at the 5% level, the positive coefficient suggests that larger firms may have a higher FP, likely due to economies of scale or resource advantages. Moreover, insignificant coefficients for L1 and L2 indicate no evidence of delayed effects of firm size on financial performance. A significant negative relationship of current leverage (−0.881) with p < 0.05indicate the impact of lower financial performance may be attributed to increased financial risk or interest burdens. while at L1 and L2 significant positive effect of leverage implies that leveraging decisions from earlier periods contribute to improved financial performance, likely reflecting strategic investments financed by debt. The diagnostic test of Arellano-Bond Test for Serial Correlation, AR(2): z = −0.89 with p > 0.05, indicating no second-order autocorrelation, validating the use of lagged variables as instruments. The value of chi^2^(151) = 34.89 with p > 0.05 of Hansen Test indicates that the valid instruments.

The model 2 results presents that the GI was regressed on corporate governance. The significant and positive coefficient (β = 0.448, p < 0.01) indicates that governance structures encourage green innovation in businesses. The coefficient of the first lag of innovation is negative and statistically significant (−0.157, p < 0.01), indicating a persistence effect. This suggests that innovation has a diminishing effect over time, where past levels of innovation negatively influence current innovation activities. Firms might face resource constraints or diminishing returns from prior investments in innovation. The contemporaneous effect CG is insignificant with p > 0.05 highlighting that the CG does not have any direct impact on innovation argument supported by study findings of [46]. Moreover, the first lag (L1) CG is also insignificant with p > 0.05. while, the second lag has positive and significant influence with p < 0.01, indicating that governance takes time to impact on green innovation. These results aligns with the findings of [49] where the top management commitment supports innovation. This delayed effect highlights the long-term importance of CG in thriving GI. Arellano-Bond Tests for Serial Correlation highlight that both AR(1) and AR(2) with reported p = 0.303 and p = 0.348 respectively highlighting no first-order and second-order serial correlation and validate the model specification to use a lagged variable as an instrument. Moreover, Hansen’s test with p = 1.000 highlights, used instruments are valid and not overidentified.

The model 3 results indicates that performance was significantly and favourably impacted by GI (β = 0.258, p < 0.01), indicating that companies with more robust green innovation practices typically have better financial results. This finding is consistent with earlier studies [58,92,93] that innovation driven by sustainability adds to the long-term value of the company. The contemporaneous effect of GI is negative and significant (−0.078, p < 0.01) and L1 is also negative and significant with p < 0.01. This implies that more innovation activities might have an immediate cost or resource constraint, thus affecting short-term profitability.the study results align with the findings of study [94]. The Arellano-Bond Tests for Serial Correlation of AR(2) is insignificant (p = 0.413), confirming no second-order serial correlation, validate the model specification and appropriateness of using lagged variables as instruments. The Hansen Test reports insignificant (p = 1.000), confirming that the instruments are valid and robust.

The present research is an attempt to look at the impact of corporate governance on firm performance through an indirect way of corporate green (GI) innovation. The diagnostic test of Model 4 confirms the validity of the GMM estimation. The Arellano–Bond test revealed significant first-order serial correlation (AR(1), p = 0.012) but no evidence of second-order serial correlation (AR(2), p = 0.293), meeting the key assumptions of the model. The Hansen J-test supported the validity of the instruments (χ^2^(183) = 33.44, p = 1.000), although the Sargan test was significant (p = 0.007), likely due to its sensitivity to heteroskedasticity and instrument count. Overall, these diagnostics suggest that the model is correctly specified and the instruments used are appropriate [70,80]. The results in Table 7 reveal that green innovation acts as a key channel through which corporate governance influences firm performance, with evidence of partial mediation emerging at the second lag.

H4 (Indirect): CG → GI → FP (supported)

To evaluate the robustness of the study’s core findings the industry-specific fixed effects regressions for key sectors—Oil & Gas, Cement, and Energy was performed. This analysis accounts for unobserved sector-level heterogeneity and tests whether the relationships between corporate governance (CG), green innovation (GI), and firm performance (FP) measured by return on assets, are consistent across different industrial contexts.

The results of sensitivity analysis presented in Table 8 indicates that industry-level estimations were broadly consistent with the full-sample findings in terms of direction and significance of effects. However, some variation in coefficient magnitudes across sectors was observed, highlighting sectoral heterogeneity in how governance and innovation practices translate into performance outcomes. This aligns with previous studies that emphasize the role of industry-specific institutional and operational factors in shaping the effectiveness of corporate governance mechanisms [95,96].

**Table 8:** Sensitivity analysis.

Variable	Gas Distribution	cement	Pharmaceuticals	Oil&Gas	Chemical
CG	−5.187** (1.534)	−3.950*** (1.222)	1.717* (2.426)	−2.953* (1.691)	−0.531* (1.745)
GI	5.154** (1.74)	5.183*** (1.777)	7.557** (3.33)	−4.296 (0.763)	−2.84** (1.34)
size	5.98*** (0.865)	5.176* (3.094)	13.951*** (4.611)	6.628*** (2.342)	18.46* (7.82)
leverage	–	−4.99** (1.455)	−4.661*** (1.384)	−2.823** (1.265)	−4.608 (4.418)
_cons	−11.661 (2.000)	−6.034 (6.761)	−27.774 (1.384)	−11.013* (5.29)	−37.87* (19.40)
R squared	0.89	0.35	0.69	0.21	0.71

*** p < 0.01, ** p < 0.05, * p < 0.1.

To ensure robustness, sensitivity analysis via industry-specific fixed-effects (FE) regressions was analyzed. Subsample estimations for key sectors—Gas Distribution, Cement, Pharmaceuticals, Oil & Gas, and Chemicals—demonstrated varying magnitudes and directions, consistent with GMM findings. For example, CG had a consistently negative impact on FP in Gas and Cement sectors, while GI positively affected FP in most sectors except Oil & Gas. These patterns highlight sectoral heterogeneity in CG practices and innovation dynamics, consistent with prior research.

### Summary of results

**Table:** 

Hypothesis	Path	Decision
H1 (Direct)	CG → FP	Supported
H2 (Direct)	GI → FP	Supported
H3 (Direct)	CG → GI	Supported
H4 (Indirect)	CG → GI → FP	Supported

The sensitivity analysis strengthens the study’s conclusions by showing that the observed relationships are not driven solely by a specific industry or subsample. Rather, they reflect more generalizable dynamics that hold across different sectors, although with varying strengths. This approach is consistent with recommendations in the literature for robustness checks using subsample analyses to ensure the validity of panel data results [97]. These findings underscore that corporate governance and green innovation dynamics are shaped by industry-specific, institutional and operational factors.

## Discussion

The results of this study provide insights into the governance, green innovation and firm performance relationship within the context of an emerging economy. By examining the mediating role of green innovation, the results advance existing research on how corporate governance influences firm outcomes, especially in environments marked by institutional weaknesses and rising environmental challenges

First, the evidence shows that effective corporate governance structures have a direct and positive impact on financial performance. This is consistent with earlier research that highlights the importance of governance in lowering agency costs, strengthening accountability, and aligning managerial incentives with the firm’s long-term goals [7–9].The positive link between corporate governance and financial performance observed here also supports the argument of Stakeholder Theory, which stresses that firms create sustainable value by balancing the interests of multiple stakeholders [17]. By providing oversight and transparency, governance mechanisms not only mitigate managerial opportunism but also strengthen investor and stakeholder confidence, which ultimately translates into improved profitability.

Second, the results demonstrate that GI exerts a significant positive effect on FP, supporting arguments that innovation can be both an ecological necessity and a strategic opportunity [2,5,6]. Although descriptive statistics initially suggested that GI may carry short-term performance costs. The GI negative impact on performance is reported in study of [98] due to implicit and explicit cost. The panel and dynamic estimations revealed that these investments yield long-term performance benefits. This finding conform with earlier work suggesting that innovation enhances operational efficiency, reputation, and market expansion [62–64]. The findings also highlighted that in emerging economies such as Pakistan, where environmental challenges are acute [21,22], green innovation (GI) is not only socially desirable but also economically viable.

Third, CG significantly influence the adoption of Green innovation (GI), confirming its role as a critical enabler of organizational change. This result is consistent with prior studies indicating that board diversity, shareholder engagement, and performance-linked incentives drive firms toward more sustainable practices [11,14]. governance provides the structures by institutionalizing accountability and long-term orientation, which is necessary to overcome managerial risk aversion and resource constraints. While, encouraging a culture supportive of innovation. At the same time, the findings echo earlier arguments that excessively rigid governance can limit creativity [15]. This highlights the need for governance mechanisms to strike a balance between control and flexibility in order to unlock the full potential of innovation.

Fourth, the mediation analysis shows that green innovation serves as a key channel through which governance translates into stronger performance. This provides empirical support for the link among corporate governance, green innovation, and financial performance, thereby extending the existing literature by validating this triadic relationship within the context of an emerging economy. While, prior studies have examined the direct impacts of governance on performance [12,13]. The present study demonstrates how governance indirectly contributes to organizational performance by fostering green innovation. This highlights a nuanced pathway through which firms can achieve sustainable competitive advantages, offering theoretical refinement to Stakeholder Theory. These findings indicates that how stakeholder aligned governance promotes not only compliance but also proactive innovation.

The findings suggest that the mediating role of green innovation (GI) between corporate governance (CG) and firm performance (FP) differs across time horizons. In the short run, the direct effect of CG on FP is statistically insignificant, while the indirect path through GI remains significant. This indicates that firms rely primarily on innovation-related strategies to translate governance structures into performance gains, demonstrating a case of full mediation. However, when lagged effects are considered, a different pattern emerges. At lag 2, CG shows a significant positive association with both GI and FP, and GI in turn exerts a positive effect on FP. This evidence reflects a partial mediation effect in the long run, where CG directly enhances FP while also indirectly improving it through GI. These results reinforce the argument that the benefits of governance systems on performance in emerging economies may not materialize immediately but require time to be channeled through innovation-driven initiatives [73,74,77,79]. The evidence also highlights the dual nature of GI as in short-term investments in green innovation may initially burden firms, over time they contribute positively to financial outcomes, aligning corporate governance with sustainable growth objectives.

Finally, industry-level analysis shows that the relationship between corporate governance, green innovation, and firm performance is not uniform across sectors. Variations were evident in industries such as Oil & Gas, Cement, and Pharmaceuticals, suggesting that institutional and operational contexts strongly shape the effectiveness of governance mechanisms and innovation strategies. For example, sectors characterized by heavy regulation or high capital intensity tend to face stricter pressures and constraints compared to more consumer-oriented industries. These differences highlight the importance of designing sector-specific governance frameworks that align with the environmental and strategic realities of each industry.

The current study extends current literature by deepening the understanding of how governance influences performance through the mediating role of green innovation in an emerging economy. Theoretically, it contributes by linking Stakeholder Theory with the dynamic pathways of innovation, while empirically it adds evidence from Pakistan, an emerging economy, a context that has received relatively little attention in prior research. From a practical standpoint, the findings suggest that governance reforms can be a powerful driver of sustainable innovation, which ultimately strengthens long-term financial performance.

## Conclusion

This research explored the nexus between corporate governance, green innovation, and financial performance in the setting of an emerging economy, with particular emphasis on the mediating role of green innovation. Methodologically, it applied a dynamic panel-data estimation approach, specifically the two-step difference GMM, to address challenges such as endogeneity, simultaneity, and dynamic heterogeneity. The analysis revealed that the governance–innovation–performance link is multifaceted and time-dependent, offering new insights for both academics and practitioners.

The findings demonstrate that corporate governance affects financial performance directly and indirectly, the latter through its influence on green innovation. While green innovation may initially weigh on short-term profitability due to costs and risks, these investments can deliver substantial long-term competitive advantages. This dual effect reinforces the importance of adopting a forward-looking, sustainable perspective when allocating resources to innovation. The results also shed light on the role of leverage, showing that its impact on performance is context-dependent: it can either constrain or enhance outcomes depending on how strategically it is managed.

Theoretically, this research makes important contributions by incorporating green innovation as a mediator, thereby extending agency and stakeholder theories to better capture the ways in which governance practices shape financial outcomes. It also enriches the debate on innovation strategy by highlighting the temporal and strategic dimensions of green innovation. Furthermore, the study demonstrates the effectiveness of dynamic panel-data models in exploring intricate causal relationships in finance and management research.

From a practical standpoint, the study offers actionable recommendations for emerging economies. Companies should develop effective governance frameworks that actively promote green innovation to address climate challenges and align with the UN sustainable goals. Boards and management must recognize that innovation’s economic returns may be delayed, necessitating strategies that balance short-term risks with long-term rewards. Effective leverage management is crucial to ensure firms can invest in green innovation without compromising their financial stability. Investors and stakeholders should look beyond financial metrics and consider green innovation and governance quality when determining sustainable growth and firm value.

Despite its contributions, the study has limitations. The small sample size and focus on specific firms listed in emerging economy stock market may limit the generalizability of the findings. While the GMM approach addresses endogeneity issues, some residual biases may remain. Additionally, the short time horizon and certain unobserved external factors calls further consideration. Future research could extend the temporal scope to overcome these limitations, utilize larger and more diverse samples, and examine the interplay between governance, green innovation, and performance across various industries and countries. Moreover, subsequent studies may incorporate moderating variables such as uncertainty, regulatory frameworks, or the extent of digital transformation to develop a more comprehensive understanding of the relationship between governance and green innovation.

## References

[1] Meeting coverage & press releases. United Nations. 2020. https://www.un.org/en/meetings/coverage-press-releases

[2] Barakat B, Milhem M, Naji GMA, Alzoraiki M, Muda HB, Ateeq A, et al. Assessing the Impact of Green Training on Sustainable Business Advantage: Exploring the Mediating Role of Green Supply Chain Practices. Sustainability. 2023;15(19):14144. doi: 10.3390/su151914144

[3] Bekun FV, Ozturk I. Economic globalization and ecological impact in emerging economies in the post‐COP21 agreement: A panel econometrics approach. Wiley Online Library. 2024.

[4] Huo J, Meng J, Zheng H, Parikh P, Guan D. Achieving decent living standards in emerging economies challenges national mitigation goals for CO2 emissions. Nat Commun. 2023;14(1):6342. doi: 10.1038/s41467-023-42079-8 37816741 PMC10564770

[5] Alofaysan H, Radulescu M, Balsalobre-Lorente D, Si Mohammed K. The effect of eco-friendly and financial technologies on renewable energy growth in emerging economies. Heliyon. 2024;10(17):e36641. doi: 10.1016/j.heliyon.2024.e36641 39281578 PMC11395751

[6] Wei X, Ren H, Ullah S, Bozkurt C. Does environmental entrepreneurship play a role in sustainable green development? Evidence from emerging Asian economies. Economic Research-Ekonomska Istraživanja. 2022;36(1):73–85. doi: 10.1080/1331677x.2022.2067887

[7] Al-ahdal WM, Alsamhi MH, Tabash MI, Farhan NHS. The impact of corporate governance on financial performance of Indian and GCC listed firms: An empirical investigation. Research in International Business and Finance. 2020;51:101083. doi: 10.1016/j.ribaf.2019.101083

[8] Aslam E, Haron R. Does corporate governance affect the performance of Islamic banks? New insight into Islamic countries. CG. 2020;20(6):1073–90. doi: 10.1108/cg-11-2019-0350

[9] Bobillo AM, Rodríguez‐Sanz JA, Tejerina‐Gaite F. Corporate governance drivers of firm innovation capacity. Rev International Economics. 2017;26(3):721–41. doi: 10.1111/roie.12321

[10] Agere S. Promoting good governance: Principles, practices and perspectives. Commonwealth Secretariat. 2000.

[11] Chang C-S, Yu S-W, Hung C-H. Firm risk and performance: the role of corporate governance. Rev Manag Sci. 2014;9(1):141–73. doi: 10.1007/s11846-014-0132-x

[12] Alodat AY, Salleh Z, Hashim HA. Corporate governance and sustainability disclosure: evidence from Jordan. CG. 2022;23(3):587–606. doi: 10.1108/cg-04-2022-0162

[13] Makpotche M, Bouslah K, M’Zali B. Corporate governance and green innovation: international evidence. RAF. 2024;23(2):280–309. doi: 10.1108/raf-04-2023-0137

[14] Lin B, Xie Y. Impacts of digital transformation on corporate green technology innovation: Do board characteristics play a role?. Corp Soc Responsibility Env. 2023;31(3):1741–55. doi: 10.1002/csr.2653

[15] Zahra SA, Neubaum DO, Huse M. Entrepreneurship in Medium-Size Companies: Exploring the Effects of Ownership and Governance Systems. Journal of Management. 2000;26(5):947–76. doi: 10.1177/014920630002600509

[16] Tatar M, Harati J, Farokhi S, Taghvaee V, Wilson FA. Good governance and natural resource management in oil and gas resource-rich countries: A machine learning approach. Resources Policy. 2024;89:104583. doi: 10.1016/j.resourpol.2023.104583

[17] Edward Freeman R, Phillips RA. Stakeholder Theory: A Libertarian Defense. Bus Ethics Q. 2002;12(3):331–49. doi: 10.2307/3858020

[18] Bui H, Krajcsák Z. The impacts of corporate governance on firms’ performance: from theories and approaches to empirical findings. JFRC. 2023;32(1):18–46. doi: 10.1108/jfrc-01-2023-0012

[19] Farooq M, Noor A, Ali S. Corporate governance and firm performance: empirical evidence from Pakistan. CG. 2021;22(1):42–66. doi: 10.1108/cg-07-2020-0286

[20] Mishra NK, Mishra N, Sharma PP. Unraveling the relationship between corporate governance and green innovation: a systematic literature review. MRR. 2025;48(5):825–45. doi: 10.1108/mrr-09-2023-0645

[21] Khan MA. Climate change risk and reduction approaches in Pakistan. Disaster risk reduction approaches in Pakistan: Springer; 2014. 195–216.

[22] Adnan M, Xiao B, Bibi S, Xiao P, Zhao P, Wang H, et al. Known and Unknown Environmental Impacts Related to Climate Changes in Pakistan: An Under-Recognized Risk to Local Communities. Sustainability. 2024;16(14):6108. doi: 10.3390/su16146108

[23] Khanna M, Anton WRQ. Corporate Environmental Management: Regulatory and Market-Based Incentives. Land Economics. 2002;78(4):539–58. doi: 10.2307/3146852

[24] Huang X, Liu W, Zhang Z, Zhao Z. Intensive judicial oversight and corporate green innovations: Evidence from a quasi-natural experiment in China. China Economic Review. 2022;74:101799. doi: 10.1016/j.chieco.2022.101799

[25] Xie Y, Wu D, Li X, Tian S. How does environmental regulation affect productivity? The role of corporate compliance strategies. Economic Modelling. 2023;126:106408. doi: 10.1016/j.econmod.2023.106408

[26] Chen C. Development of green business strategies through green dynamic capabilities and environmental regulation: Empirical evidence from the construction sector. Journal of Cleaner Production. 2024;438:140826. doi: 10.1016/j.jclepro.2024.140826

[27] Deng F, Zhou C. Sustainable Development of Corporate Governance in the Hospitality and Tourism Industry: The Evolution and the Future. Sustainability. 2022;14(7):4286. doi: 10.3390/su14074286

[28] Chang Y, Chen T-H, Shu M-C. Corporate Social Responsibility, Corporate Performance, and Pay-Performance Sensitivity—Evidence from Shanghai Stock Exchange Social Responsibility Index. Emerging Markets Finance and Trade. 2018;54(5):1183–203. doi: 10.1080/1540496x.2016.1273768

[29] Basile V. Corporate social responsibility and social report: A case study in the Basque Country. Corporate social responsibility in the 21st century. IntechOpen. 2022.

[30] Tam OK, Tan MG. Ownership, Governance and Firm Performance in Malaysia. Corporate Governance. 2007;15(2):208–22. doi: 10.1111/j.1467-8683.2007.00555.x

[31] Khan MT, Al‐Jabri QM, Saif N. Dynamic relationship between corporate board structure and firm performance: Evidence from Malaysia. Int J Fin Econ. 2019;26(1):644–61. doi: 10.1002/ijfe.1808

[32] Hutchinson M, Gul FA. Investment opportunity set, corporate governance practices and firm performance. Journal of Corporate Finance. 2004;10(4):595–614. doi: 10.1016/s0929-1199(03)00022-1

[33] Naimah Z. The role of corporate governance in firm performance. SHS Web of Conferences. 2017.

[34] Chauhan Y, Lakshmi KR, Dey DK. Corporate governance practices, self-dealings, and firm performance: Evidence from India. Journal of Contemporary Accounting & Economics. 2016;12(3):274–89. doi: 10.1016/j.jcae.2016.10.002

[35] Javaid F, Saboor A. Impact of Corporate Governance index on Firm Performance: evidence from Pakistani manufacturing sector. jpag. 2015;5(2):1. doi: 10.5296/jpag.v5i2.7498

[36] Azeez AA. Corporate Governance and Firm Performance: Evidence from Sri Lanka. JFBM. 2015;3(1). doi: 10.15640/jfbm.v3n1a16

[37] Affes W, Jarboui A. The impact of corporate governance on financial performance: a cross-sector study. Int J Discl Gov. 2023;20(4):374–94. doi: 10.1057/s41310-023-00182-8

[38] Rebeiz KS, Salameh Z. Relationship between Governance Structure and Financial Performance in Construction. J Manage Eng. 2006;22(1):20–6. doi: 10.1061/(asce)0742-597x(2006)22:1(20

[39] Hassan Che Haat M, Abdul Rahman R, Mahenthiran S. Corporate governance, transparency and performance of Malaysian companies. Managerial Auditing Journal. 2008;23(8):744–78. doi: 10.1108/02686900810899518

[40] Kowalewski O. Corporate governance and corporate performance: financial crisis (2008). MRR. 2016;39(11):1494–515. doi: 10.1108/mrr-12-2014-0287

[41] Kyere M, Ausloos M. Corporate governance and firms financial performance in the United Kingdom. Int J Fin Econ. 2020;26(2):1871–85. doi: 10.1002/ijfe.1883

[42] Palaniappan G. Determinants of corporate financial performance relating to board characteristics of corporate governance in Indian manufacturing industry. EJMBE. 2017;26(1):67–85. doi: 10.1108/ejmbe-07-2017-005

[43] Rossi M, Chouaibi J, Chouaibi S, Jilani W, Chouaibi Y. Does a Board Characteristic Moderate the Relationship between CSR Practices and Financial Performance? Evidence from European ESG Firms. JRFM. 2021;14(8):354. doi: 10.3390/jrfm14080354

[44] Alabdullah TTY, Naseer HQ. Corporate governance strategic performance as a significant strategic management to promoting profitability: a study in UAE. JHSSB. 2023;2(4):620–35. doi: 10.55047/jhssb.v2i4.706

[45] Luo K, Zhang K. Executive’s environmental background and sustainable development: evidence from substantial green innovation. Sustainable Development. 2024.

[46] Ma Z, Shu G, Wang Q, Wang L. Sustainable Governance and Green Innovation: A Perspective from Gender Diversity in China’s Listed Companies. Sustainability. 2022;14(11):6403. doi: 10.3390/su14116403

[47] Velte P. Sustainable board governance and environmental performance: European evidence. Bus Strat Env. 2023;33(4):3397–421. doi: 10.1002/bse.3654

[48] Basile V, Loia F, Capobianco N, Vona R. An ecosystems perspective on the reconversion of offshore platforms: Towards a multi‐level governance. Corp Soc Responsibility Env. 2022;30(4):1615–31. doi: 10.1002/csr.2439

[49] Liu Z, Guo Y, Zhang M, Mao T. Smog risk perception, corporate social responsibility, and green innovation: evidence from China. SRJ. 2023;19(8):1419–34. doi: 10.1108/srj-06-2021-0249

[50] Jin H, Wang Q, Wu L. Sustainable city development from the perspective of corporate green innovation and governance. Sustainable Cities and Society. 2024;102:105216. doi: 10.1016/j.scs.2024.105216

[51] Fan Y, Su Q, Wang X, Fan M. Digitalization and green innovation of enterprises: Empirical evidence from China. Front Environ Sci. 2023;11. doi: 10.3389/fenvs.2023.1120806

[52] Li J, Wang L, Nutakor F. Empirical research on the influence of corporate digitalization on green innovation. Front Environ Sci. 2023;11. doi: 10.3389/fenvs.2023.1137271

[53] Wang A, Si L, Hu S. Can the penalty mechanism of mandatory environmental regulations promote green innovation? Evidence from China’s enterprise data. Energy Economics. 2023;125:106856. doi: 10.1016/j.eneco.2023.106856

[54] Bai X, Lyu C. Executive’s Environmental Protection Background and Corporate Green Innovation: Evidence from China. Sustainability. 2023;15(5):4154. doi: 10.3390/su15054154

[55] Schiederig T, Tietze F, Herstatt C. Green innovation in technology and innovation management – an exploratory literature review. R & D Management. 2012;42(2):180–92. doi: 10.1111/j.1467-9310.2011.00672.x

[56] Yin X, Wang D, Lu J, Liu L. Does green credit policy promote corporate green innovation? Evidence from China. Econ Change Restruct. 2023;56(5):3187–215. doi: 10.1007/s10644-023-09521-9

[57] Farza K, Ftiti Z, Hlioui Z, Louhichi W, Omri A. Does it pay to go green? The environmental innovation effect on corporate financial performance. J Environ Manage. 2021;300:113695. doi: 10.1016/j.jenvman.2021.113695 34649325

[58] Wang YZ, Ahmad S. Green process innovation, green product innovation, leverage, and corporate financial performance; evidence from system GMM. Heliyon. 2024;10(4):e25819. doi: 10.1016/j.heliyon.2024.e25819 38390127 PMC10881324

[59] Aguilera-Caracuel J, Ortiz-de-Mandojana N. Green Innovation and Financial Performance. Organization & Environment. 2013;26(4):365–85. doi: 10.1177/1086026613507931

[60] Ai M, Luo F, Bu Y. Green innovation and corporate financial performance: Insights from operating risks. Journal of Cleaner Production. 2024;456:142353. doi: 10.1016/j.jclepro.2024.142353

[61] Lestari ER, Sunyoto NMS. Fostering green innovation in achieving sustainable performance. Wiley Online Library. 2023.

[62] Wang J, Xue Y, Sun X, Yang J. Green learning orientation, green knowledge acquisition and ambidextrous green innovation. Journal of Cleaner Production. 2020;250:119475. doi: 10.1016/j.jclepro.2019.119475

[63] Chen W, Zhu C, Cheung Q, Wu S, Zhang J, Cao J. How does digitization enable green innovation? Evidence from Chinese listed companies. Bus Strat Env. 2024;33(5):3832–54. doi: 10.1002/bse.3672

[64] Wang C, Yan G, Ou J. Does Digitization Promote Green Innovation? Evidence from China. Int J Environ Res Public Health. 2023;20(5):3893. doi: 10.3390/ijerph20053893 36900903 PMC10001908

[65] Yuan B, Cao X. Do corporate social responsibility practices contribute to green innovation? The mediating role of green dynamic capability. Technology in Society. 2022;68:101868. doi: 10.1016/j.techsoc.2022.101868

[66] Chang C-H. The Influence of Corporate Environmental Ethics on Competitive Advantage: The Mediation Role of Green Innovation. J Bus Ethics. 2011;104(3):361–70. doi: 10.1007/s10551-011-0914-x

[67] Chang C. The Determinants of Green Product Innovation Performance. Corp Soc Responsibility Env. 2014;23(2):65–76. doi: 10.1002/csr.1361

[68] Qiu L, Jie X, Wang Y, Zhao M. Green product innovation, green dynamic capability, and competitive advantage: Evidence from Chinese manufacturing enterprises. Corp Soc Responsibility Env. 2019;27(1):146–65. doi: 10.1002/csr.1780

[69] Wu S, Zhou X, Zhu Q. Green credit and enterprise environmental and economic performance: The mediating role of eco-innovation. Journal of Cleaner Production. 2023;382:135248. doi: 10.1016/j.jclepro.2022.135248

[70] Ozkan A, Temiz H, Yildiz Y. Climate Risk, Corporate Social Responsibility, and Firm Performance. British J of Management. 2022;34(4):1791–810. doi: 10.1111/1467-8551.12665

[71] Chouaibi S, Chouaibi J, Rossi M. ESG and corporate financial performance: the mediating role of green innovation: UK common law versus Germany civil law. EMJB. 2021;17(1):46–71. doi: 10.1108/emjb-09-2020-0101

[72] Rauf F, Wanqiu W, Naveed K, Zhang Y. Green R & D investment, ESG reporting, and corporate green innovation performance. PLoS One. 2024;19(3):e0299707. doi: 10.1371/journal.pone.0299707 38547119 PMC10977761

[73] Arellano M, Bond S. Application to Employment Equations. 1991.

[74] Blundell R, Bond S. Initial conditions and moment restrictions in dynamic panel data models. Journal of Econometrics. 1998;87(1):115–43. doi: 10.1016/s0304-4076(98)00009-8

[75] Sharma R, Mehta K, Goel A. Non-linear relationship between board size and performance of Indian companies. J Manag Gov. 2022;27(4):1277–301. doi: 10.1007/s10997-022-09651-8

[76] Co. C. ESG challenges in emerging markets. 2024. https://www.clydeco.com/en/insights/2024/08/esg-challenges-in-emerging-markets

[77] Bellstam G, Bhagat S, Cookson JA. A Text-Based Analysis of Corporate Innovation. Management Science. 2021;67(7):4004–31. doi: 10.1287/mnsc.2020.3682

[78] Lu Q, Chesbrough H. Measuring open innovation practices through topic modelling: Revisiting their impact on firm financial performance. Technovation. 2022;114:102434. doi: 10.1016/j.technovation.2021.102434

[79] Nousiainen E, Ranta M, Ylinen M, Järvenpää M. Using machine learning and 10‐K filings to measure innovation. Accounting & Finance. 2024;64(4):3211–39. doi: 10.1111/acfi.13245

[80] Dutta S, Kumar A, Pant P, Walsh C, Dutta M. Using 10-K text to gauge COVID-related corporate disclosure. PLoS One. 2023;18(3):e0283138. doi: 10.1371/journal.pone.0283138 36947527 PMC10032508

[81] Yang N. Corporate Risk Information Disclosure Based on Semantic Analysis Methods. Mobile Information Systems. 2022;2022:1–13. doi: 10.1155/2022/9381918

[82] Chen Y-S, Lai S-B, Wen C-T. The Influence of Green Innovation Performance on Corporate Advantage in Taiwan. J Bus Ethics. 2006;67(4):331–9. doi: 10.1007/s10551-006-9025-5

[83] Liang D, Lu C-C, Tsai C-F, Shih G-A. Financial ratios and corporate governance indicators in bankruptcy prediction: A comprehensive study. European Journal of Operational Research. 2016;252(2):561–72. doi: 10.1016/j.ejor.2016.01.012

[84] Chen C, Chen C, Lien D. Financial distress prediction model: The effects of corporate governance indicators. Journal of Forecasting. 2020;39(8):1238–52. doi: 10.1002/for.2684

[85] Roodman D. How to Do xtabond2. In: 2006.

[86] Preacher KJ, Hayes AF. Asymptotic and resampling strategies for assessing and comparing indirect effects in multiple mediator models. Behav Res Methods. 2008;40(3):879–91. doi: 10.3758/brm.40.3.879 18697684

[87] Wintoki MB, Linck JS, Netter JM. Endogeneity and the dynamics of internal corporate governance. Journal of Financial Economics. 2012;105(3):581–606. doi: 10.1016/j.jfineco.2012.03.005

[88] Roodman D. How to do Xtabond2: An Introduction to Difference and System GMM in Stata. The Stata Journal: Promoting communications on statistics and Stata. 2009;9(1):86–136. doi: 10.1177/1536867x0900900106

[89] Baron RM, Kenny DA. The moderator-mediator variable distinction in social psychological research: conceptual, strategic, and statistical considerations. J Pers Soc Psychol. 1986;51(6):1173–82. doi: 10.1037//0022-3514.51.6.1173 3806354

[90] Yan Q, Yan J, Zhang D, Bi S, Tian Y, Mubeen R, et al. Does CEO Power Affect Manufacturing Firms’ Green Innovation and Organizational Performance? A Mediational Approach. Sustainability. 2024;16(14):6015. doi: 10.3390/su16146015

[91] Cavaco S, Crifo P, Guidoux A. Corporate Social Responsibility and Governance: The Role of Executive Compensation. Industrial Relations. 2020;59(2):240–74. doi: 10.1111/irel.12254

[92] Cheng B, Ioannou I, Serafeim G. Corporate social responsibility and access to finance. Strategic Management Journal. 2013;35(1):1–23. doi: 10.1002/smj.2131

[93] Maldonado-Guzmán G, Garza-Reyes JA, Pinzón-Castro SY. Green innovation and firm performance: the mediating role of sustainability in the automotive industry. Management of Environmental Quality: An International Journal. 2023;34(6):1690–711.

[94] Zhang Z, Duan H, Shan S, Liu Q, Geng W. The Impact of Green Credit on the Green Innovation Level of Heavy-Polluting Enterprises-Evidence from China. Int J Environ Res Public Health. 2022;19(2):650. doi: 10.3390/ijerph19020650 35055471 PMC8776069

[95] Aggarwal R, Erel I, Stulz R, Williamson R. Differences in Governance Practices between U.S. and Foreign Firms: Measurement, Causes, and Consequences. Rev Financ Stud. 2008;22(8):3131–69. doi: 10.1093/rfs/hhn107

[96] Haniffa R, Hudaib M. Corporate Governance Structure and Performance of Malaysian Listed Companies. Business Fin & Account. 2006;33(7–8):1034–62. doi: 10.1111/j.1468-5957.2006.00594.x

[97] Chenhall RH, Moers F. The Issue of Endogeneity within Theory-Based, Quantitative Management Accounting Research. European Accounting Review. 2007;16(1):173–96. doi: 10.1080/09638180701265937

[98] Li Y, Armstrong A, Clarke A. The relationship between corporate governance and financial performance of small corporations in Australia. JBSGE. 2014;9(2). doi: 10.15209/jbsge.v9i2.716

